# Infographic of the Ocular Hypertension Study (OHTS)

**DOI:** 10.1038/s41433-020-01370-4

**Published:** 2021-06-11

**Authors:** Syed Mustafa Ali Ahmad, Ji Eun Han, Rashmi G. Mathew, Christin Henein

**Affiliations:** UCL Institute of Ophthalmology, London, UK

**Keywords:** Glaucoma, Education

**Fig. 1:**
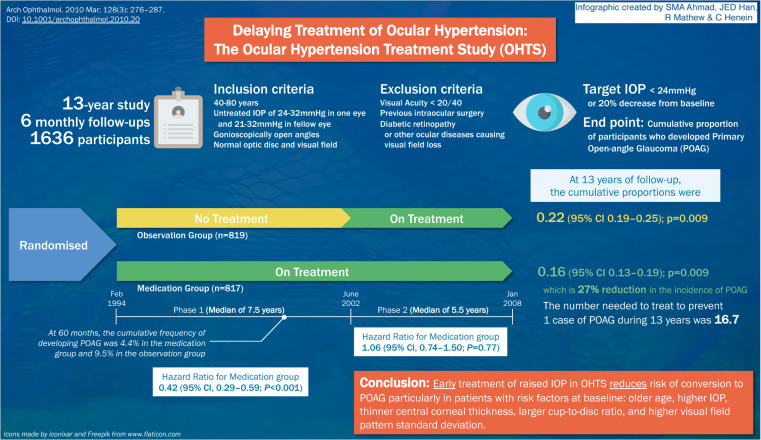
The Ocular Hypertension Study (OHTS) showed that early treatment of raised intraocular pressures in patients with ocular hypertension reduced the risk of conversion to primary open angle glaucoma, particularly in patients with baseline risk factors. IOP intraocular pressure, POAG primary open angle glaucoma, CI confidence interval.

