# Correction: Infographic of the Ocular Hypertension Study (OHTS)

**DOI:** 10.1038/s41433-021-01691-y

**Published:** 2021-07-19

**Authors:** Syed Mustafa Ali Ahmad, Ji Eun Han, Rashmi G. Mathew, Christin Henein

**Affiliations:** UCL Institute of Ophthalmology, London, UK

**Keywords:** Glaucoma, Education

Correction to: *Eye* 10.1038/s41433-020-01370-4, published online 11 June 2021

Unfortunately, the middle initial was missing in author’s name Rashmi G. Mathew.

The original article has been corrected.

